# The Application of the Generalized Additive Model to Represent Macrobenthos near Xiaoqing Estuary, Laizhou Bay

**DOI:** 10.3390/biology12081146

**Published:** 2023-08-18

**Authors:** Lulei Liu, Ang Li, Ling Zhu, Suyan Xue, Jiaqi Li, Changsheng Zhang, Wenhan Yu, Zhanfei Ma, Haonan Zhuang, Zengjie Jiang, Yuze Mao

**Affiliations:** 1 College of Fisheries and Life Science, Shanghai Ocean University, Shanghai 201306, China; a18142072420@163.com; 2 Yellow Sea Fisheries Research Institute, Chinese Academy of Fishery Sciences, Qingdao 266071, China; liang19990217@163.com (A.L.); zhuling@ysfri.ac.cn (L.Z.); xuesy@ysfri.ac.cn (S.X.); lijq@ysfri.ac.cn (J.L.); 82101202455@caas.cn (C.Z.); yuwh97@163.com (W.Y.); mzfegb@126.com (Z.M.); zhuanghnan@163.com (H.Z.); jiangzj@ysfri.ac.cn (Z.J.); 3 Laboratory for Marine Ecology and Environmental Science, Laoshan Laboratory, Qingdao 266237, China; 4 School of Fishery, Zhejiang Ocean University, Zhoushan 316022, China

**Keywords:** GAM, application, macrobenthos, Laizhou Bay

## Abstract

**Simple Summary:**

Macrobenthos is widely used as an indicator of ecological health in marine monitoring and assessment. Xiaoqing Estuary has been subjected to different degrees of freshwater injection, resulting in significant changes in salinity, which has affected macrobenthos in the area. In this paper, we determined the main environmental variables driving the macrobenthic community and established a generalized additive model (GAM) model of macrobenthos based on the Margalef diversity index (*d*_M_), following which the distribution of macrobenthos richness can be predicted. The present findings will provide useful information for future studies on the correlation between macrobenthic communities and environmental factors in salinity-stressed areas of estuaries.

**Abstract:**

Macrobenthos is widely used as an indicator of ecological health in marine monitoring and assessment. The present study aimed to characterize the interrelationships between the distribution of the macrobenthos community and environmental factors near Xiaoqing Estuary, Laizhou Bay. Responses of species richness to environmental factors were studied using the generalized additive model (GAM) and the Margalef diversity index (*d*_M_) as indicators of species diversity instead of individual indicator species. Six factors were selected in the optimal model by stepwise regression: sediment factors (organic matter, phosphate, nitrate nitrogen, and ammonium nitrogen) and water factors (salinity, and ammonium nitrogen). The response curves generated by the GAM showed a unimodal relationship among taxa diversity, salinity in water, and sediment organic matter. *d*_M_ was positively correlated with ammonium nitrogen in water and was negatively correlated with phosphate in the sediment. The model optimized by forward stepwise optimization explained 92.6% of the Margalef diversity index with a small residual (2.67). The model showed good performance, with the measured *d*_M_ strongly correlated with the predicted *d*_M_ (Pearson R^2^ = 0.845, *p* < 0.05). The current study examined the combined influence of multiple eco-factors on macrobenthos, and the Margalef diversity index of macrobenthos was predicted by the GAM model in a salinity-stressed estuary.

## 1. Introduction

Macrobenthos is widely used as an indicator of ecological health in marine monitoring and assessment due to the relatively weak ability of macrobenthos species to migrate, their long life cycles, and their differential tolerance to multiple stressors [1,2,3]. Macrobenthos communities also play a key role in the functioning of estuarine systems, which connect freshwater and oceans and are important spawning and feeding grounds for marine organisms [4]. Since estuaries are easily disturbed by anthropogenic activities, the ecological status of benthic organisms is regularly assessed in estuaries and adjacent areas [5]. There have been increasing anthropogenic pressures on coastal habitat, including coastal development and habitat degradation [6]. Consequently, there has been a decline in the biodiversity of macrobenthos due to aquatic ecosystem habitat loss and degradation [7]. In addition, the spatial and temporal distributions of the estuarine biological community may be directly or potentially affected by the changes in water and sedimentary environments [8]. Benthic communities are directly affected by a variety of physical and chemical environmental factors, including temperature, salinity, hydrodynamic status, sediment type and particle size, and nutrient content [9,10,11,12]. Therefore, there is a need to explore the habitat requirements of macrobenthos communities and their responses to changes in environmental factors.

Ecologists have attempted to explore the effects of different environmental conditions on macrobenthos communities by developing a variety of models or statistical methods suitable for understanding the community- and population-scale distributions of environmental factors [13,14]. The generalized linear model (GLM) can be used to fit regression models for univariate response data. The GLM relates a function of the mean to environmental variables through a prediction equation of a linear form. The GAM is an expansion and a nonparametric modification of GLM, which is itself a generalization of multiple linear regression (MLR) and is not only able to screen various environmental factors and fit the best model but can also intuitively evaluate the relationships between the macrobenthos community and various environmental factors in the form of a graph. It has the ability to deal with the different types of distributions that characterize ecological data [15]. Since the relationships between organisms and environmental factors are very complex, linear regression is not appropriate to study these relationships. Consequently, the nonlinear analysis methods of the GLM and GAM models have been increasingly used to investigate these relationships. The advantage of the GAM over the traditional regression method relates to its ability to integrate multiple environmental variables within a quantitative evaluation of the influence and importance of each factor [16]. The GAM has also been widely used in the study of the relationships between fishery resources and environmental factors [17,18].

Previous studies have examined disturbances to macrobenthos and changes to community structure due to the impacts of fishery activities [19]. The relationships between macrobenthic communities and ecological factors are usually nonlinear and highly complex, and it is often difficult to express these relationships using traditional mathematical equations [20]. Salinity is generally considered to be the main environmental driver of estuarine function and community dynamics. Seasonal freshwater inputs and precipitation have been shown to reduce the abundance of macrobenthos [21]. Recent applications of GAM models have mainly focused on natural river ecosystems [22]. In contrast, there has been insufficient focus on the impact of habitat factors on macrobenthic communities in estuaries, particularly in salinity-stressed areas near Xiaoqing Estuary. Xiaoqing Estuary has been subjected to different degrees of freshwater injection, resulting in significant changes in salinity, which has affected aquaculture in the area. A previous study on macrobenthos in Xiaoqing Estuary focused only on the occurrence and composition of macrobenthos [23]. To date, there has been no comprehensive analysis of the correlation between macrobenthos and environmental factors in Xiaoqing Estuary. Therefore, the present study adopted the macrobenthos community of Xiaoqing Estuary as a case study.

Laizhou Bay is in the southern Bohai Sea, China, and is the largest bay in Shandong Province. The bay is an important fishery spawning and feeding ground in the Bohai Sea. The Xiaoqing River is the second largest river flowing into Laizhou Bay after the Yellow River. This river is a large-scale artificial river with navigation, irrigation, and sewage effluent disposal functions [24]. The Xiaoqing River imports large quantities of organic matter and nutrients into Laizhou Bay [25]. Inputs of salt to this area in September and October 2021 changed due to significant rainfall and runoff. The Xiaoqing Estuary is one of the main clam production areas in Laizhou Bay. The clam species that are farmed in this area include *Ruditapes philippinarum*, *Mactra veneriformis*, *Meretrix meretrix*, and *Cyclina sinensis*, with an annual output of ~20,000 tons.

The aim of the present study was to evaluate the correlation between environmental factors and macrobenthic communities in the salinity-stressed area near Xiaoqing Estuary. The specific objectives of the present study were to (1) characterize the composition of the macrobenthic community in the salinity-stressed area; (2) determine the main environmental variables driving the macrobenthic community; and (3) establish a GAM model of macrobenthos based on the *d*_M_, following which the distribution of macrobenthos richness can be predicted. The present study can act as a reference for future studies on the correlation between macrobenthic communities and environmental factors in salinity-stressed areas of estuaries.

## 2. Materials and Methods

### 2.1. Study Area

**Figure 1:**
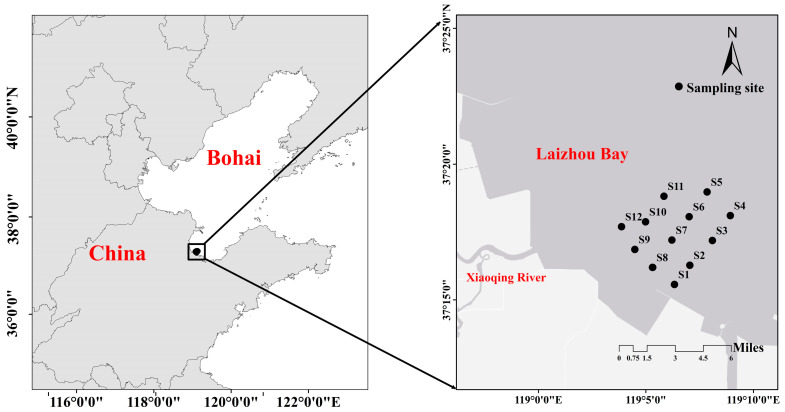
Locations of the sampling sites of the present study in Xiaoqing Estuary.

The present study conducted four surveys (in March, May, August, and October 2021) to describe the distribution of the macrobenthos community and analyze the responses between macrobenthos and habitat factors in southwest Laizhou Bay. The depth ranges from 1 m to 3.5 m in the study area. The present study established 12 sampling stations (S1–S12) in the bivalve aquaculture area between longitude 37.20° and 37.35° and latitude 119.05° and 119.15° (Figure 1) (no sediment was collected for S5 and S12 in March and S8 and S9 in October due to adverse weather conditions).

### 2.2. Sampling and Data Analysis Method

Three macrobenthos subsamples were taken at each sampling station using a Van Veen grab of 0.1 m^2^. Subsamples were pooled to represent each site. Sampled sediments were washed through a metal sieve with a 0.5 mm mesh. The filtered-out specimens were then transferred to sample bottles filled with 5% formalin. The samples were identified, classified, counted, and wet-weighted (accurate to 0.0001 g) in the laboratory, and finally converted into abundance (ind./m^2^) and biomass (g/m^2^) according to the sampling area. All samples were collected, treated, and stored according to the “Specifications for oceanographic survey—Part 6: Marine biological survey (GB/T 12763.6–2007)”.

Apart from macrobenthos-related measures, the present study also conducted four surveys, during which samples were taken in triplicate from each sampling station for the measurement of seventeen parameters. The water quality parameters and physical features measured included water depth (H), water temperature (T), pH, dissolved oxygen (DO), salinity (Sal), chlorophyll-a (Chl-a), particulate organic matter (POM), phosphate (PO_4_-P), ammonium nitrogen (NH_4_-N), nitrate nitrogen (NO_3_-N), and nitrite nitrogen (NO_2_-N). The variables associated with the sediment measured in the present study included the quantity of sediment organic matter (SOM), median particle diameter (D_50_), sediment phosphate (PO_4_-P_soil_), sediment ammonium nitrogen (NH_4_-N_soil_), sediment nitrate nitrogen (NO_3_-Nsoil), and sediment nitrite nitrogen (NO_2_-N_soil_). Water samples were collected on the surface with 1 L prelabeled plastic containers at each station. Some parameters (H, T, pH, DO, and Sal) were measured in the field using a multiparameter water quality analyzer (Smartroll Mp, Fort Collins, CO, USA). Sediments were sampled using a Van Veen grab of 0.1 m^2^ at all sampling stations. Each sediment sample was fully mixed and placed into sealed plastic bags. Water and sediment samples were collected and returned to the laboratory for the measurement of all other factors using methods corresponding to national standards [26]. The median particle diameter (D_50_) was examined by a laser particle sizer (Mastersizer 3000, Malvern City, UK). Nutrient concentrations in water and sediment were determined by an automatic nutrient fluid analyzer (Auto Analyzer Three, Bran Luebbe, Hamburg, Germany) based on “The specifications for oceanographic survey—Part 4: Seawater analysis (GB 17378.4–2007)”. Precipitation data were accessed from the ERA5 dataset (https://www.ecmwf.int/en/forecasts/datasets/reanalysis-datasets/era5) on 7 April 2022, which was generated by the fifth-generation ECMWF atmospheric reanalysis of the global climate covering the period from January 1950 to present.

Species richness is an integrative descriptor of the community. The Margalef diversity index reflects the species richness of the community, which performs well in distinguishing differences between communities. The present study used the Margalef diversity index (*d*_M_) to describe the diversity of benthic macroinvertebrates at each sampling station for each survey. Species-level taxa were used for calculating the index. *d*_M_ was calculated as [27]:

(1) $$d_{M}=\left(S-1\right)/\ln N$$

In Equation (1), *S* is taxa richness, i.e., the number of taxa within a sampling area, and *N* is the total number of individuals.

The dominant species of macrobenthos were described by the dominance index; the dominance index (*Y*) was calculated as [28]:

(2) $$Y=\left(n_{i}/N\right)\times f_{i}$$

In Equation (2), *n_i_* is the total abundance of the *i*-th species at all stations, *N* is the total abundance of individuals at all stations, and *f_i_* is the frequency of occurrence of the *i*-th species at all stations. Species *i* is defined as dominant when *Y* > 0.02.

A GAM was developed to quantify relationships between the Margalef diversity index (*d*_M_) and key ecological factors [26]. The general form of the GAM is:

(3) $$f\left(\mu \left(N\right)\right)=\beta_{0}+Y_{1}\left(x_{1}\right)+\ldots +Y_{n}\left(x_{n}\right)$$

n=0,1,2

In Equation (3), *f*(.) is the connection function, *μ*(*N*) is the expected value of the response variable *Y*, *β*_0_ is the intercept, and *Y*_i_(.) is the smoothing function for the *i*_th_ explanatory variable *x*_i_.

We used 17 eco-factors, including H, T, pH, Sal, DO, POM, NH_4_-N, NO_3_-N, NO_2_-N, PO_4_-P, SOM, D_50_, NH_4_-N_soil_, NO_3_-N_soil_, NO_2_-N_soil_, PO_4_-P_soil_, and NO_3_-N_soil_, as explanatory variables within the construction of the GAM. The model was constructed using the data sampled in March, May, and August. First, the taxa diversity index (*d*_M_) was examined to assess the significance of the effects of the single environmental factors on *d*_M_ (a significance level of 0.01 was specified). Factors having a significant effect on *d*_M_ were retained in the model. A forward selection procedure that sequentially added variables was used. Stepwise regression was used to assess the accuracy of the model according to the Akaike Information Criterion (AIC). The AIC value of the single-factor prediction function was detected, following which other environmental factors were progressively added to the single-factor prediction function until no further decrees in the AIC could be obtained.

The present study regarded the model with the smallest AIC value to be the optimal model. The significance of the prediction model was evaluated based on the results of the F-test. The generalized additive model was implemented by the “mgcv” package in R software [16].

The similarities between sites were calculated by means of the Bray–Curtis coefficient [29] and hierarchical clustering (CLUSTER), and non-metric multidimensional scaling (NMDS) analyses were used to reveal macrofaunal assemblage groups in the sampling sites in different sites, and they were applied using PRIMER 6.0 [30]. Analysis of similarity (ANOSIM) was widely used in ecology to determine whether there was a significant difference between intergroup and intra-group distance [31], and an ANOSIM test was calculated in R using the Vegan package. The data were analyzed and plotted using Excel 2019 and R 3.6.3 statistical analysis software (Lucent Technologies, State of New Jersey, USA), and the results were presented as mean ± standard deviation. The environmental factors, biomass, and abundance of macrobenthos were analyzed using a one-way analysis of variance (ANOVA) (*p* < 0.05). Following the identification of significant differences, Duncan’s multiple comparison test was conducted to assess differences between the groups.

## 3. Results

### 3.1. Ecological Factors

**Figure 2:**
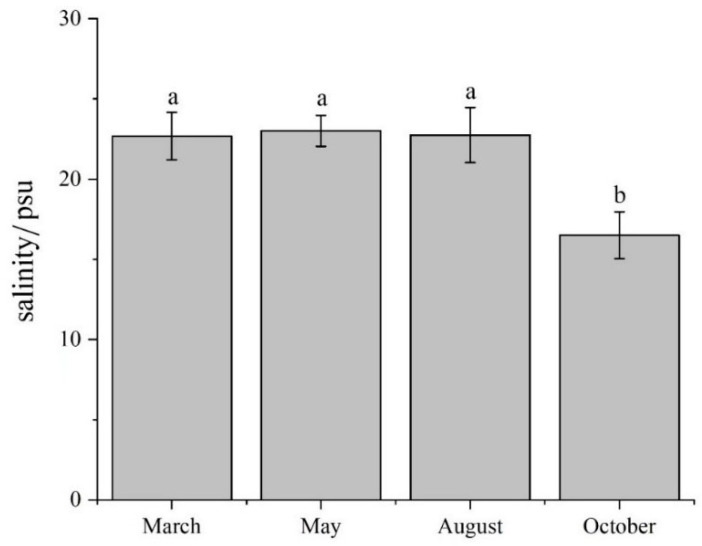
The average value of salinity in March, May, August, and October 2021, respectively (mean ± standard deviation). We used lowercase letters to indicate the significant difference between cruises. Data labeled without the same letter (s) were significantly (*p* < 0.05) different from each other.

**Figure 3:**
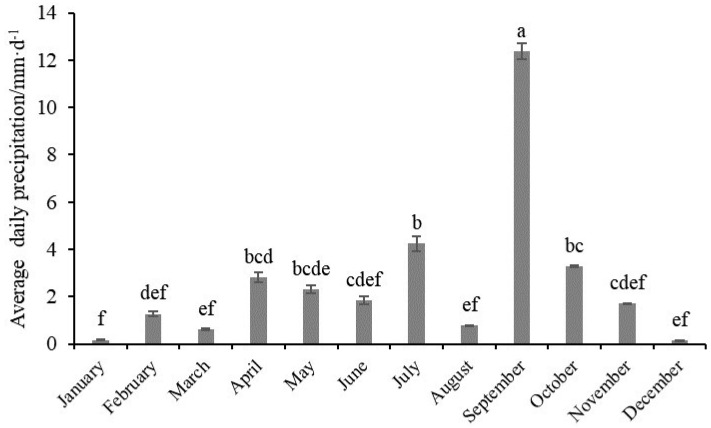
Average daily precipitation for each month in 2021 (mean ± standard deviation). The superscript letter to value indicates a significant difference at the different months (*p* < 0.05). We used lowercase letters to indicate the significant difference between different months. Data labeled without the same letter (s) were significantly (*p* < 0.05) different from each other.

There were significant differences in environmental variables in the water column among the four months, in addition to H, pH, and POM (Table 1). There were significant differences in environmental variables in the sediment over the four months. The results of the four surveys showed no significant differences in salinity at all sites in March, May, and August (*p* > 0.05). However, Sal over these months was significantly higher than in October (*p* < 0.05) (Table 1 and Figure 2). The contents of DIN, DIN_soil_, PO_4_-P, and PO_4_-P_soil_ in October were significantly higher than in the other months. The average daily precipitation in September significantly exceeded the value in other months (*p* < 0.05) (Figure 3).

### 3.2. Composition of the Macrobenthos Fauna

**Figure 4:**
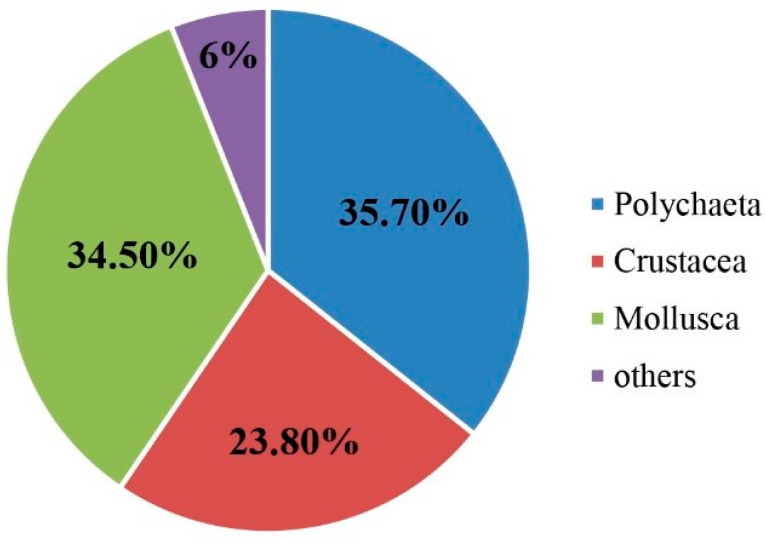
Community structure of macrobenthos over four months in 2021.

**Figure 5:**
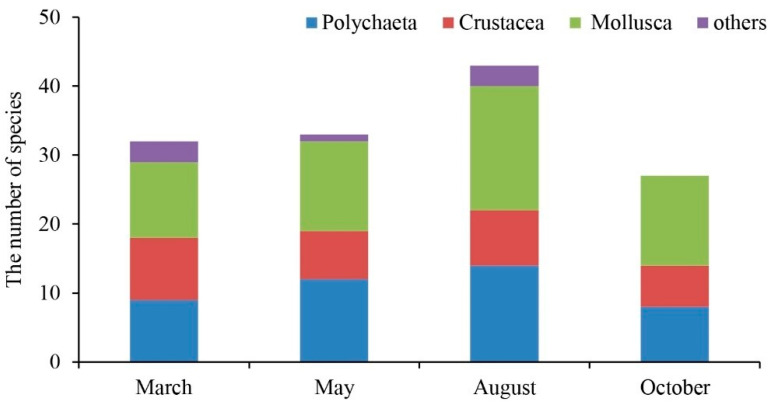
The number of macrobenthos species in March, May, August, and October 2021, respectively.

The macrobenthos taxa were classified to species level for further analysis. The four surveys obtained eighty-four macrobenthos species, including sixty-one families falling into nine phyla, nine classes, and thirty-one orders. Among these, there were 30 species of Polychaeta, including twelve orders, twenty-one families, and twenty-five genera, accounting for 35.7% of the total taxonomic unit. This was followed by twenty-nine species of Mollusca, including nine orders, twenty-one families, and twenty-four genera, accounting for 34.5% of the total taxonomic unit. There were twenty species of crustaceans, including six orders, twelve families, and fifteen genera, accounting for 23.8% of the total taxonomic unit, and five other species. There were four orders, five families, and five genera, accounting for 6% of the total taxonomic unit (Figure 4). The highest number of species was in August, whereas only polychaetes, crustaceans, and mollusks were found in October (Figure 5). In March, 32 taxa were collected, with *Mactra chinensis* and *Notomastus latericeus Sars* being the dominant taxa. In May, 33 taxa were collected, with the dominant taxa being *Mactra chinensis*, *Cultellus attenuates*, *Nephtys polybranchia*, and *Heterocuma sarst*. In August, 43 taxa were collected, with *Mactra veneriformis*, *Ruditapes philippinarum*, and *Musculus senhousei* being the dominant taxa. In October, 28 taxa were collected, with the dominant taxa being *Mactra veneriformis*, *Ruditapes philippinarum*, *Musculus senhousei,* and *Decorifera matusimana* (Figure 5 and Table 2). Overall, there were significant differences in the composition of dominant macrobenthic species between the four voyages.

**Figure 6:**
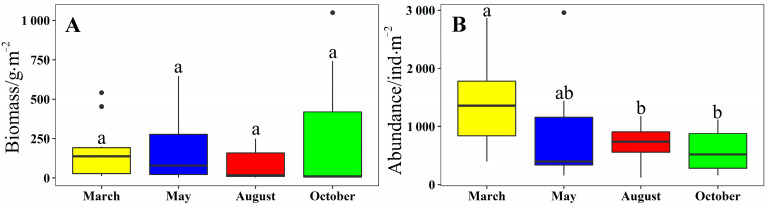
(**A**,**B**) Biomass and abundance of macrobenthos in March, May, August, and October 2021, respectively (mean ± standard deviation). We used lowercase letters to indicate the significant difference between cruises. Data labeled without the same letter (s) were significantly (*p* < 0.05) different from each other.

There were no significant differences in the biomass of macrobenthos between the different months (Kruskal–Wallis test, *p* = 0.478, Figure 6A). October and August showed the highest and lowest biomass, respectively (mean ± SD of 248.34 ± 80.39 g/m^2^ and 99.63 ± 26.98 g/m^2^, respectively). There were significant differences in the densities of macrobenthos among the different months (Kruskal–Wallis test, *p* = 0.027). October showed the lowest density at 560.30 ± 58.27 ind./m^2^ (Figure 6B). 

**Figure 7:**
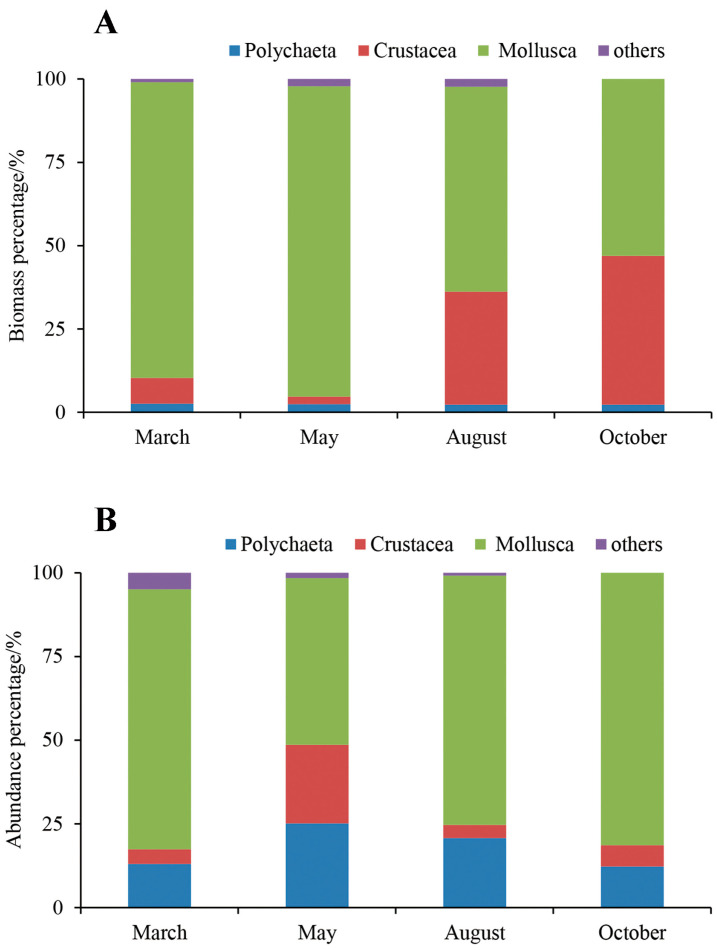
(**A**,**B**) Biomass and abundance percentage of macrobenthos in March, May, August, and October 2021, respectively.

The proportion of crustaceans in macrobenthos in October significantly exceeded those in other months (Figure 7A), which could be attributed to larger hermit crab (*Pagurus* sp.) samples from station S5. Mollusks contributed higher proportions of total macrobenthos biomass in March, May, August, and October at 89.60%, 99.10%, 62.35%, and 53.07%, respectively (Figure 7A). Mollusks showed higher contributions to macrobenthos density in March, May, August, and October at 77.71%, 49.80%, 74.42%, and 81.35%, respectively. In contrast to the other months, only polychaetes, mollusks, and crustaceans were observed in October (Figure 7B).

### 3.3. The Community Structure of Macrobenthos

**Figure 8:**
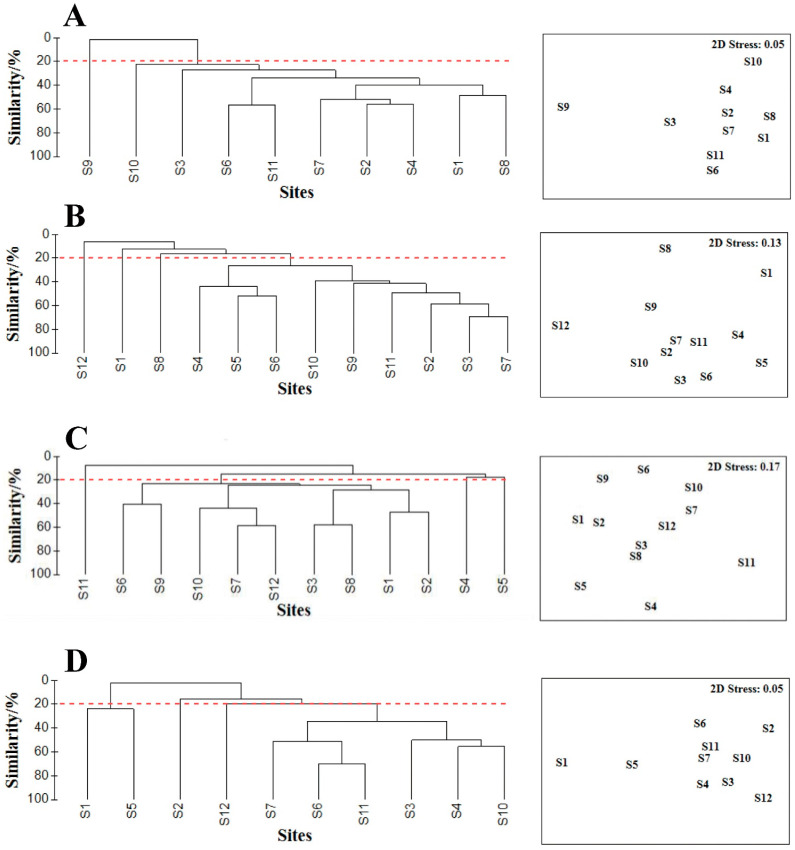
(**A**–**D**) The results of cluster analysis and non-metric multidimensional scaling (NMDS) for macrobenthos in March, May, August, and October 2021, respectively. Notes: the dashed line represents the level of similarity in dividing the community structure.

The Bray–Curtis coefficient indicated low similarities between macrobenthos communities at each station between the four voyages of 20%. The results of the cluster analysis were consistent with those of the non-metric multidimensional scaling (NMDS) analysis in different months (Figure 8). All stations could be divided into two communities in March at a similarity level of 20%, and four communities in May, August, and October, respectively, at a similarity level of 20%. NMDS indicated the stress coefficients in March, May, August, and October to be 0.05, 0.13, 0.17, and 0.05, respectively. The results of ANOSIM showed significant differences between the different cluster groups in four surveys (March, R = 0.9259, *p* < 0.01; May, R = 0.7685, *p* < 0.01; August, R = 0.5789, *p* < 0.01; October, R = 0.8707, *p* < 0.01).

### 3.4. Responses of Community Diversity to Ecological Factors

**Figure 9:**
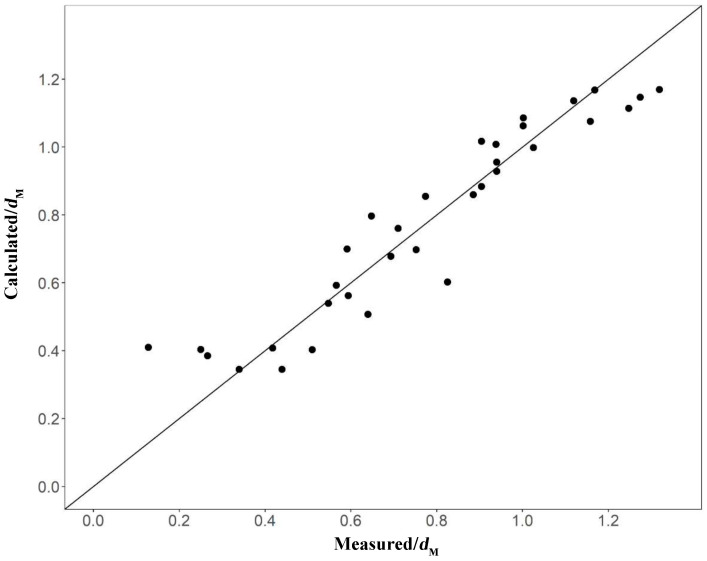
Calculated Margalef diversity index (*d*_M_) versus *d*_M_ based on Model 9.

Twelve environmental factors that had significant effects on taxa diversity (*d*_M_) were identified (H, T, pH, Sal, DO, POM, SOM, NH_4_-N, PO_4_-P_soil_, NH_4_-N_soil_, NO_3_-N_soil_, and D_50_). Since the correlation coefficients between DO and NH_4_-N and between T and NH_4_-N_soil_ exceeded 0.5, these variables were not simultaneously added to the model to avoid collinearity, and higher correlations between *d*_M_ and NH_4_-N, NH_4_-N_soil_, and Sal were retained. The remaining nine environmental indicators were used to establish the GAM, and the model structure was optimized by forward stepwise regression (Table 3). The addition of the variables SOM, PO_4_-P_soil_, NO_3_-N_soil_, NH_4_-N_soil_, Sal, and NH_4_-N significantly increased model performance (*p* < 0.05). Variables H, T, and pH were removed since their inclusion did not improve model performance. Model 9 represents the final form of the model: *d*_M_ ~ s (SOM) + s (PO_4_-P_soil_) + s (NO_3_-N_soil_) + s (NH_4_-N_soil_) + s (Sal) + s (NH_4_-N). The model explained 92.6% of the variance (adjusted coefficient of determination R^2^ = 0.845). Pearson’s correlation coefficient between the calculated *d*_M_ and the measured *d*_M_ was highly correlated at 0.9635 (*p* < 0.05, Figure 9).

**Figure 10:**
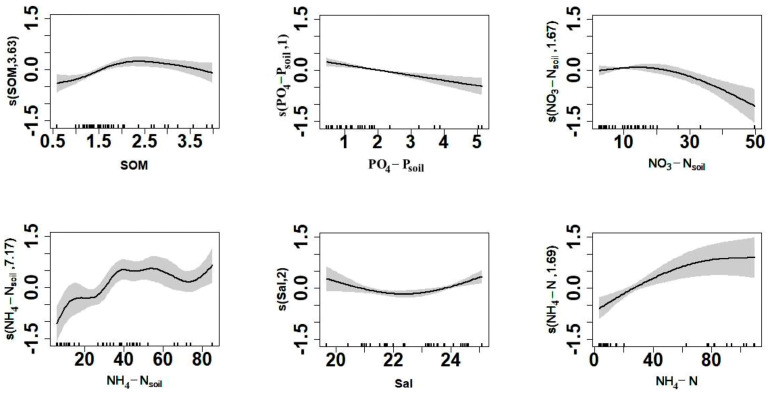
Response curves of the Margalef diversity index (*d*_M_) to ecological factors in the generalized additive model (GAM) analysis.

The response curve of *d*_M_ to environmental factors shows a unimodal relationship between species diversity, SOM, and Sal. In addition, there was a linear relationship between species diversity and NH_4_-N and PO_4_-P_soil_, and *d*_M_ was positively correlated with NH_4_-N and negatively correlated with PO_4_-P_soil_ (Figure 10).

### 3.5. Validation of the Model

**Figure 11:**
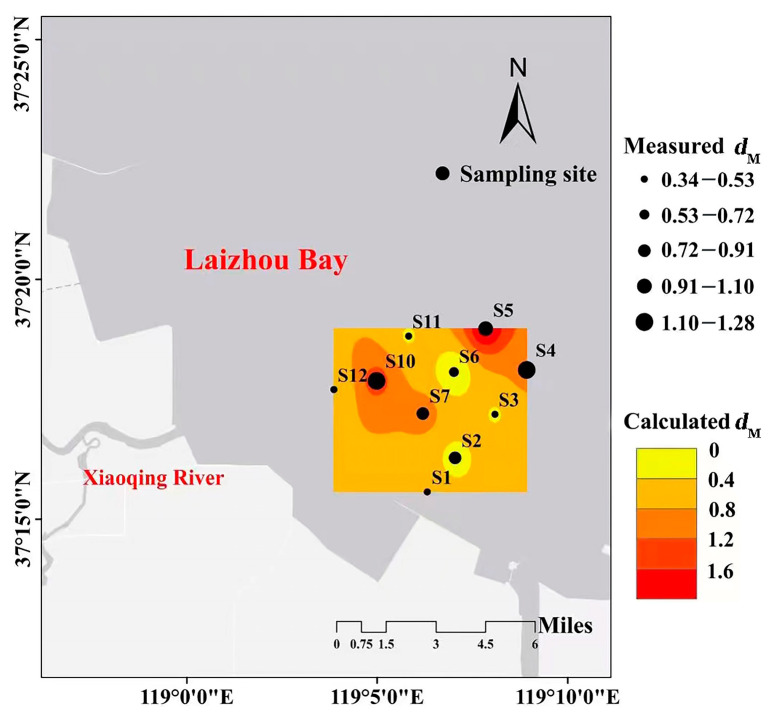
The spatial distributions of the measured and predicted Margalef diversity index (*d*_M_) in October 2021.

Sampling data collected in October 2021 were used to validate Model 9. As shown in Table 4, measured *d*_M_ values were strongly correlated with the *d*_M_ predicted by Model 9 (Pearson R^2^ = 0.845, *p* < 0.05), with a small mean squared error (MSE). This result confirmed the good performance of the model and its ability to effectively simulate the distribution of benthic fauna diversity in the bivalve farming and salinity-stressed area in the Xiaoqing estuary, Laizhou Bay (Figure 11).

## 4. Discussion

The salinity of the study area in October was significantly lower than the three other investigated months (*p* < 0.05). This result could possibly be attributed to heavy precipitation. The hydrological data indicated that the precipitation dilution of salinity increased significantly in September. Previous studies have shown that heavy rainfall can result in a significant short-term increase in river runoff, which not only reduces the salinity of surface water in Laizhou Bay but also facilitates the transportation of large quantities of nutrients of terrestrial origin into the Bay. This input of nutrients, in turn, leads to an increase in nutrient concentrations (dissolved inorganic nitrogen and phosphate) in the sea area [32,33]. Consistent with the above point, the present study determined that the contents of dissolved inorganic nitrogen and phosphate in surface and interstitial water were significantly higher in October than in other months (Table 1).

The survey identified 84 species of macrobenthos. The order of the major taxonomic groups in terms of species numbers was polychaeta > mollusks > crustaceans. This result is consistent with a previous study [34]. The results of the present study indicated that species number and abundance in October were lower than in other months, which was possibly related to lower salinity. Some previous studies have shown that salinity is a vital environmental factor affecting the distribution of macrobenthic species in estuarine areas. Salinity concentration shows an inverse relationship to macrobenthic species abundance [23,35]. The structure of the macrobenthic community may be influenced by unstable environmental factors, particularly the influence of river runoff and rainfall changes on the marine environment. In addition, studies in other sea areas have shown that the complex marine environment results in the formation of different habitat niches, which are inhabited by different benthic community structures, resulting in low similarities between the benthic communities of each station [36,37]. Mollusks had larger contributions to biomass over the four months due to their relatively larger body sizes.

The stress coefficient of the NMDS analysis was less than 0.2, which reflected the relationships among species within the macrobenthos community in each month. The results of the clustering and non-metric multidimensional scale analysis indicated a low similarity of macrobenthos communities in the survey area, which is consistent with the results of previous studies [19,23]. Despite the differences in the community structure of macrobenthos between the various months, the model showed a total residual deviation after optimization of 2.67 and an AIC of −20.54. Pearson’s correlation coefficient between the calculated and measured *d*_M_ was high at 0.9635 (*p* < 0.05). This result was consistent with a previous study, which showed that the GAM performed well in predicting the *d*_M_ of macrobenthos under unchanging salinity in October.

Since the comprehensive multiparameter evaluation index relies heavily on the weights of parameters, the biological index is more suitable for the assessment of ecological health under the influences of anthropogenic activities [20]. Identifying the spatiotemporal distribution of the benthic community is essential for the conservation and sustainable development of local benthic resources. The relationships between various environmental factors, such as temperature and salinity, on benthic species richness are often not linear. However, the GAM typically shows higher performance in analyzing the nonlinear relationship between dependent and multiple independent variables. Thus, the application of the GAM has great significance for the study of benthic communities. The results of the GAM analysis showed that each factor had different effects on the changes to the macrobenthic abundance index in the coastal waters of Xiaoqing River Estuary in Laizhou Bay, and the relationships among them were mostly nonlinear. The present study screened and fitted the response curves of the macrobenthic richness index to key environmental factors based on the GAM. The results of the GAM indicated that environmental variables (Sal, SOM, NH_4_-N, PO_4_-P_soil_, NH_4_-N_soil_, and NO_3_-N_soil_) had the greatest influence on the benthic community in the study area. Organic matter and nutrients are often the factors limiting the survival of benthic communities [38,39]. Salinity is an important environmental factor affecting the survival, growth, and distribution of benthic communities [40]. The salinity of the benthic environment ranged between 21 and 25 psu in March, May, and August. The model results showed a minimum *d*_M_ under a salinity of 22.5 psu. The rate of decline in *d*_M_ was highest under a salinity of 20–22 psu, whereas there was a gradually increasing trend at a salinity of 24–25 psu. The GAM indicated that *d*_M_ was positively correlated with NH_4_-N and negatively correlated with PO_4_-P_soil_, which was consistent with the results of previous studies. NH_4_-N is an essential nutrient for the growth of aquatic plants and algae in water, and the application of nitrogen to aquatic plants in previous studies improved the productivity of macrobenthos [22,41]. Eutrophication results from increases in PO_4_-P_soil_ increase the biomass and diversity of plankton but result in changes to the community structure and a reduction in the species richness of benthic communities [42]. There were high correlations between DO and NH_4_-N, T, and NH_4_-N_soil_, with increases in T and DO. Although there were clear changes in NH_4_-N and NH_4_-N_soil_, the factors T and DO were removed to increase the degree of fit of the model. Other studies have suggested that DO is an important factor regulating the survival of benthos, with impacts on the abundance and distribution of macrobenthos [43]. Therefore, the present study considered the interactions between these environmental factors, and the above environmental factors were added to the GAM to allow comprehensive future studies.

The distribution of target species was predicted by exploring the relationship between species distribution and related variables using the species distribution model. The GAM has been widely used to explore relationships between species distribution and environmental factors in fish and submerged plants [44,45]. However, there have been few studies on the relationships between macrobenthos and environmental variables in estuaries. The present study applied the GAM in combination with the common zero-value richness index to analyze the distribution of benthic resources in the bivalve farming and salinity-stressed estuary area, Laizhou Bay. Due to the relationship between the mean of the response variable and a smoothed function of the predictor variables, which was established by a link function, a parametric function of the model was not produced as one potential drawback of the GAM. But, we can make predictions based on the model [15]. In addition, the present study did not consider the influences of spatial and temporal auto-correlation on the modeling. Future studies can improve the accuracy of the model by considering the effect of time through the addition of an autoregressive process.

It was also worth noting that the establishment of the GAM model was based on environmental data surveyed in March, May, and August 2021. Although salinity in October was significantly lower than in March, May, and August, the distribution of benthic diversity was effectively predicted by the GAM model at each site in October. The GAM model was able to predict the species richness of the macrobenthos when salinity was significantly lower in October. Therefore, the present study provides a preliminary exploration of the relationships between the macrobenthic Margalef diversity index and environmental factors. Future studies should apply different methods (such as the habitat index, linear partial differential equation with first-order variable coefficient, and quantile regression) to integrate long-term quantitative and environmental data into future habitat suitability models. These models can then be used to more comprehensively analyze the distribution and dynamics of benthic organisms. Moreover, there have been changes to some environmental factors in the study area, such as salinity and inorganic salts, due to heavy rain, which may partially explain the deviation in the model results.

## 5. Conclusions

Among the seventeen environmental factors investigated in the present study, sediment factors (organic matter, phosphate, nitrate nitrogen, and ammonium nitrogen) and water factors (salinity and ammonium nitrogen) were the key factors affecting the structure and diversity of the macrobenthic community in the coastal waters of Xiaoqing Estuary. The response curve of *d*_M_ to environmental factors shows unimodal relationships between species diversity and sediment organic matter and between species diversity and salinity. In addition, there were linear relationships between species diversity and ammonium nitrogen and between species diversity and phosphate in interstitial water, and *d*_M_ was positively and negatively correlated with ammonium nitrogen and sediment phosphate, respectively. The optimal GAM model explained 92.6% of the observed variation in the macrobenthic Margalef diversity index with a small residual (2.67). The measured *d*_M_ was strongly correlated with the predicted *d*_M_ (Pearson R^2^ = 0.845, *p* < 0.05). In general, the model showed good performance and could effectively simulate the distribution of benthic fauna diversity in the salinity-stressed area in Xiaoqing Estuary in Laizhou Bay. The complementary use of different indices is recommended to assess the richness of macrobenthos in China.

## Figures and Tables

**Table 1:** Values for each of the measured ecological factors in March, May, August, and October 2021, respectively.

	Factors	March	May	August	October
Water	H/m	1.89 ± 0.26 ^a^	3.07 ± 0.78 ^b^	3.40 ± 0.76 ^b^	3.00 ± 0.51 ^b^
	T/°C	9.33 ± 0.85 ^a^	16.94 ± 0.73 ^b^	27.70 ± 0.82 ^c^	14.60 ± 0.85 ^d^
	pH	7.94 ± 0.15 ^a^	7.96 ± 0.05 ^a^	7.94 ± 0.05 ^a^	7.81 ± 0.11 ^b^
	DO/mg·L^−1^	8.76 ± 0.43 ^a^	7.62 ± 0.92 ^a^	5.96 ± 0.40 ^b^	8.47 ± 0.30 ^a^
	Sal/psu	22.70 ± 1.22 ^a^	23.02 ± 0.97 ^a^	22.75 ± 1.72 ^a^	17.01 ± 1.67 ^b^
	Chl-a/μg·L^−1^	4.66 ± 0.46 ^a^	4.53 ± 1.57 ^a^	5.31 ± 1.82 ^a^	4.85 ± 1.67 ^a^
	POM/mg·L^−1^	8.42 ± 0.60 ^a^	8.83 ± 1.93 ^a^	7.79 ± 1.29 ^a^	7.39 ± 2.40 ^a^
	PO_4_-P/μmol·L^−1^	0.17 ± 0.03 ^a^	0.22 ± 0.10 ^a^	2.23 ± 1.10 ^b^	2.63 ± 0.98 ^c^
	NH_4_-N/μmol·L^−1^	7.88 ± 1.13 ^a^	8.34 ± 5.31 ^a^	39.43 ± 5.82 ^b^	95.24 ± 25.22 ^c^
	NO_3_-N/μmol·L^−1^	28.39 ± 8.84 ^a^	32.13 ± 7.26 ^a^	33.90 ± 10.80 ^a^	25.70 ± 7.52 ^a^
	NO_2_-N/μmol·L^−1^	3.50 ± 1.09 ^a^	6.05 ± 2.62 ^a,b^	4.06 ± 1.56 ^a,b^	1.65 ± 0.84^b^
	DIN/μmol·L^−1^	39.77 ± 10.57 ^a^	46.52 ± 12.28 ^a^	77.39 ± 12.15 ^b^	122.59 ± 31.00 ^c^
Sediment	SOM/%	1.86 ± 0.91 ^a^	1.92 ± 0.53 ^a^	1.99 ± 0.93 ^a^	2.85 ± 1.59 ^b^
	D_50_/μm	89.58 ± 15.00 ^a^	78.80 ± 24.70 ^a^	72.53 ± 34.52 ^a^	74.17 ± 20.50 ^a^
	PO_4_-P_soil_/μmol·L^−1^	0.96 ± 0.34 ^a^	2.13 ± 1.25 ^a,b^	2.89 ± 1.35 ^a,b^	3.66 ± 1.22 ^b^
	NH_4_-N_soil_/μmol·L^−1^	4.07 ± 0.68 ^a^	10.41 ± 4.49 ^a^	36.05 ± 11.11 ^b^	55.34 ± 24.97 ^c^
	NO_3_-N_soil_/μmol·L^−1^	3.94 ± 1.13 ^a^	13.54 ± 3.08 ^b^	17.90 ± 12.61 ^b,c^	23.18 ± 9.32 ^c^
	NO_2_-N_soil_/μmol·L^−1^	0.42 ± 0.09 ^a^	1.37 ± 0.46 ^b^	3.26 ± 0.90 ^c^	2.90 ± 1.60 ^c^
	DIN_soil_/μmol·L^−1^	8.43 ± 1.29 ^a^	25.32 ± 6.04 ^b^	57.20 ± 20.50 ^c^	81.42 ± 32.73 ^d^

Notes: DIN: dissolved inorganic nitrogen; DIN_soil_: dissolved inorganic nitrogen in sediment. We used lowercase letters to indicate the significant difference between cruises. Data labeled without the same letter (s) were significantly (*p* < 0.05) different from each other.

**Table 2:** Dominant species of macrobenthos in March, May, August, and October 2021, respectively.

Group	Species	Dominance			
		March	May	August	October
Mollusca	*Mactra chinensis*	0.536	0.157		
	*Mactra veneriformis*			0.021	0.223
	*Ruditapes philippinarum*			0.065	0.047
	*Musculus senhousei*			0.214	0.023
	*Decorifera matusimana*				0.026
	*Cultellus attenuates*		0.025		
Annelida	*Nephtys polybranchia*		0.024		
	*Notomastus latericeus Sars*	0.021			
Arthropoda	*Heterocuma sarst*		0.078		

**Table 3:** Variance analysis table of the forward stepwise regression process.

Model	Residual Deviation	Cumulative Deviation	AIC	*p*
Model 1	8.4899	19.5%	28.9733	0.0035
Model 2	7.7887	27.5%	27.3027	0.0014
Model 3	6.8724	43.4%	21.9143	0.0054
Model 4	4.5450	79%	3.5953	0.9830
Model 5	4.5103	79.6%	3.1832	0.7660
Model 6	4.4361	80.2%	2.6007	0.7332
Model 7	4.3078	80.7%	3.4475	0.0002
Model 8	2.9922	90.8%	-13.9273	0.0092
Model 9	2.6667	92.6%	-20.5398	0.0081

Notes: AIC: Akaike Information Criterion. Only SOM was added to Model 1. The factors PO_4_-P_soil_, NO_3_-N_soil_, pH, POM, and water depth were successively added to Models 2, 3, 4, 5, and 6. Models 4, 5, and 6 were not improved after the addition of pH, POM, and H (*p* > 0.05). pH, POM, and H were removed from Model 7. Models 7, 8, and 9 were improved after the addition of NH_4_-N_soil_, Sal, and NH_4_-N (*p* < 0.05).

**Table 4:** Statistical summary of the performance of the optimal model (Model 9) in October 2021.

Site	Measured *d*_M_	Predicted *d*_M_	Site	Measured *d*_M_	Predicted *d*_M_
S1	0.3467	0.5320	S6	0.5758	0.1054
S2	0.8873	0.3592	S7	0.8546	0.9453
S3	0.3775	0.4296	S10	1.2792	1.2764
S4	1.2792	0.8639	S11	0.4293	0.4261
S5	1.0193	1.8208	S12	0.3941	0.4438
*MSE*	0.1363	*R* ^2^	0.845	*p*	0.0383

Notes: Data shown with mean squared error (MSE), correlation coefficient (R^2^), and significance level (*p*-value) between predicted and measured data; no sediment samples were taken at sites 8 and 9 due to adverse weather.

## Data Availability

Not applicable.
